# Optoelectrical Properties of Hexamine Doped-Methylammonium Lead Iodide Perovskite under Different Grain-Shape Crystallinity

**DOI:** 10.3390/nano13071281

**Published:** 2023-04-05

**Authors:** Marjoni Imamora Ali Umar, Annisa Zahra Ahdaliza, Salah M. El-Bahy, Nur Aliza, Siti Naqiyah Sadikin, Jaenudin Ridwan, Abang Annuar Ehsan, Mohammed A. Amin, Zeinhom M. El-Bahy, Akrajas Ali Umar

**Affiliations:** 1 Department of Physics Education, Faculty of Tarbiyah, Universitas Islam Negeri Mahmud Yunus, Batusangkar 27213, West Sumatera, Indonesia; 2 Department of Chemistry, Turabah University College, Taif University, P.O. Box 11099, Taif 21944, Saudi Arabia; 3 Institute of Microengineering and Nanoelectronics, Universiti Kebangsaan Malaysia, UKM, Bangi 43600, Selangor, Malaysia; 4 Department of Chemistry, College of Science, Taif University, P.O. Box 11099, Taif 21944, Saudi Arabia; 5 Department of Chemistry, Faculty of Science, Al-Azhar University, Nasr City, Cairo 11884, Egypt

**Keywords:** crystallinity, grain morphology, carrier dynamic, perovskite solar cells, annealing treatment

## Abstract

The crystallinity properties of perovskite influence their optoelectrical performance in solar cell applications. We optimized the grain shape and crystallinity of perovskite film by annealing treatment from 130 to 170 °C under high humidity (relative humidity of 70%). We found that the grain size, grain interface, and grain morphology of the perovskite are optimized when the sample was annealed at 150 °C for 1 h in the air. At this condition, the perovskite film is composed of 250 nm crystalline shape grain and compact inter-grain structure with an invincible grain interface. Perovskite solar cells device analysis indicated that the device fabricated using the samples annealed at 150 °C produced the highest power conversion efficiency, namely 17.77%. The open circuit voltage (V_oc_), short-circuit current density (*J*_sc_), and fill factor (FF) of the device are as high as 1.05 V, 22.27 mA/cm_2_, and 0.76, respectively. Optoelectrical dynamic analysis using transient photoluminescence and electrochemical impedance spectroscopies reveals that (i) carrier lifetime in the champion device can be up to 25 ns, which is almost double the carrier lifetime of the sample annealed at 130 °C. (ii) The interfacial charge transfer resistance is low in the champion device, i.e., ~20 Ω, which has a crystalline grain morphology, enabling active photocurrent extraction. Perovskite’s behavior under annealing treatment in high humidity conditions can be a guide for the industrialization of perovskite solar cells.

## 1. Introduction

Perovskite solar cells (PSC) have become the focus of investigation around the world due to their high performance in converting light into electrical energy, rivaling that of monocrystalline solar cells [1,2,3], and far overcoming other types of solar cells, including organic solar cells and hetero-junction solar cells [4,5]. The optoelectrical properties of the perovskite material, which are easy to change through compositional engineering [6,7], have increased the opportunity to continuously improve the performance of light to electrical energy conversion [8,9]. Efficiencies as high as over 25% have been reported so far, a level that surpasses that of monocrystalline Si solar cells. Therefore, continuous efforts to further improve PSC performance should be continued through various approaches, such as compositional engineering, interface engineering, and so on.

Despite all the approaches being found effective for improving the optoelectrical properties of perovskite, the crystallinity properties, such as stoichiometry, phase crystallinity, and grain size, play the biggest impact on the PSC performance [10,11,12]. Recently, we have discovered a new composition to produce humidity-resistant perovskite (methylammonium lead iodide), which was achieved through doping with hexamine molecules [13]. This new perovskite is very stable against humidity up to 95%. Furthermore, it can be prepared in ambient conditions, a condition that promises the process of industrialization of PSC. Based on our previous studies, we found that although hexamine was able to increase the chemical stability of perovskite [14], it caused an increase in power hysteresis and an abnormal J-V curve. Modification of the crystallinity properties of perovskite through an annealing process may be able to improve existing deficiencies. Here, we report the relationship between the crystallinity properties of perovskite and the optoelectrical properties of a hexamine doped-MAPbI_3_ and its performance in PSC. The crystallinity properties of perovskite were modified via annealing treatment. We found that besides modifying its crystallinity properties, the annealing treatment also changed the optical band edge of the perovskite material. However, we discovered that there was a non-linear relationship between crystallite size and PSC performance, where a large crystallite size, namely perovskite annealed at 170 °C, did not produce the best PSC solar cells, but instead, the best solar cells were produced from perovskite annealed at 150 °C, namely perovskite with medium-grain size. A power conversion efficiency as high as over 17% is obtained from this process. The band gap analysis found that the champion PSC has an optical band gap of around 1.5 eV. The band gap increases with increasing annealing temperature. This indicates that the difference in the conduction band energy level between the perovskite and the electron transport layer is the critical aspect that plays a major role in device performance. Thus, refining the crystallinity properties of the perovskite while maintaining the conduction band offset value with the ETL is necessary to obtain a PSC with high power conversion efficiency.

## 2. Experimental Details

### 2.1. Perovskite Solar Cells Preparations

The perovskite solar cells (PSC) fabricated in this study were using methylammonium lead triiodide (MAPbI_3_) perovskite as the absorber layer. An ambient-stable MAPbI_3_ perovskite was prepared using our recently developed method [15,16,17]. PSC with n-i-p type was fabricated using the structure of ITO/SnO_2_/MAPbI_3_/P3HT/Au. Initially, the ITO substrate was carefully cleaned by ultrasonication in 30% Decon 90 solution for 30 min and followed by two times ultrasonication in de-ionized water for 10 min each. After that, the ITO was then ultrasonicated two times in ethanol for 10 and 15 min, respectively. Finally, the ITO substrate was dried with nitrogen gas flow. 

Next, the SnO_2_ electron transport layer (ETL) was deposited on the ITO substrate by a two-step spin-coating method, i.e., 3000 rpm for 10 s and followed by 5000 rpm for 20 s. An amount of 80 µL of SnO_2_ colloidal solution was spread on the top of the ITO substrate and then spin-coated. After that, the sample was heated on a hotplate with a temperature of 180 °C for 1 h. Before the perovskite layer preparation, the SnO_2_ ETL was cleaned in isopropyl alcohol (IPA) by ultrasonication for 3 min. The sample was then dried with nitrogen gas flow and heated at 50 °C for 3 min. 

The perovskite layer on top of ETL was then prepared. The MAPbI_3_ perovskite layer was prepared by first preparing the following two solutions: (i) the PbI_2_ and (ii) methylammonium iodide (MAI) solutions. PbI_2_ solution was prepared in a glass vial with a concentration of 700 mg/mL in a mixed solvent of dimethylformamide (DMF) and dimethyl sulfoxide (DMSO) with a ratio of 8:2. The glass vial was then tightly capped and wrapped with parafilm to isolate the solution. The solution was heated to 60 °C while stirred at 700 rpm for at least 3 h. Meanwhile, MAI was prepared in IPA with a concentration of 60 mg/mL. After that, 1 mL of 3 mg/mL hexamine (hexamethylenetetramine) in the IPA was added to the MAI solution. The mixed solution was then aged at room temperature for 3 h while stirred at 700 rpm. All the processes were performed in the gloves box with a humidity of more or less 35%. The Poly(3-hexylthiophene-2,5-diyl) (P3HT) solution, which is employed to fabricate the hole transport layer (HTL), is also prepared. A total of 20.52 mg of P3HT was used in this study and dissolved in 1 mL chlorobenzene, producing a concentration of 0.38 mM. P3HT was dissolved using ultrasonic for 30 min and then aged for 3 h before use. All solutions were filtered before use.

An amount of 80 µL of PbI_2_ solution was evenly spread on top of the SnO_2_ layer and then spin-coated at 2200 rpm for 1 min. After that, the sample was heated on a hot plate at 100 °C for 1 min. The homogenous yellow color of PbI_2_ will be formed on the substrate surface. The sample was then cooled down to room temperature. Then, 80 µL of fresh IPA was evenly dropped on top of the PbI_2_ layer and spin-coated with the rotational speed of 2200 rpm for 1 min. The sample was then again heated at 100 °C for 1 min. The IPA treatment will make the PbI_2_ layer glossy. Finally, 80 µL of MAI solution was dropped on top of the PbI_2_ layer using the dynamic spin coating method at a speed of 2200 rpm for 30 s. The MAI was dropped in the spin-coating running for 5 to 10 s. The sample was then annealed for 15 min on a hot plate at 150 °C. Glossy black-color perovskite was obtained in this stage. To obtain different crystallinity properties of perovskite, the sample was annealed at temperatures varying from 130, 150, 160, and 170 °C.

After the sample cooled down to room temperature, the P3HT hole transport layer was deposited on top of the perovskite layer. The amount of 60 µL of P3HT solution was spin-coated at a speed of 3000 rpm. Finally, an Au electrode as thick as 80 nm was deposited on top of the P3HT layer via the thermal evaporation method. 

### 2.2. Perovskite Film Characterizations

Phase crystallinity of the perovskite layer was verified via X-ray diffraction spectroscopy using BRUKER D8 Advance with CuKα irradiation (λ = 1.5 Å). The optical properties of the sample were analyzed using a UV-Vis Winlab optical spectrophotometer. The photoluminescence properties of the sample were characterized by steady and transient spectroscopy using Edinburgh Instrument Model FLS920. The morphologies of the sample were evaluated through field-emission scanning electron microscopy using the FESEM Carl Zeiss Supra 55VP apparatus. 

### 2.3. Photovoltaic Performance Analysis

The PSC device’s performance was evaluated via the J-V curve analysis under the illumination of a 100 mW/cm^2^ AM1.5G solar simulator light source (Newport low-cost simulator) using a Gamry Interface 1000 potentiostat. The measurement was carried out with a scan rate of 100 mV/s. The intensity of the light source was calibrated using Tenmars TM-207 solar power meter (Taipei, Taiwan), with an accuracy of 5%. The temperature of the sample during the measurement was ca. 40 °C. For the J-V analysis, we used a metallic mask with an opening area of 0.4 cm × 0.5 cm. The electrochemical impedance spectroscopy (EIS) used to probe the carrier transport in the device also used the same potentiostat and light source. During the EIS measurement, the device was biased at V_oc_. The entire measurement was carried out in the ambient condition.

## 3. Results and Discussions

**Figure 1:**
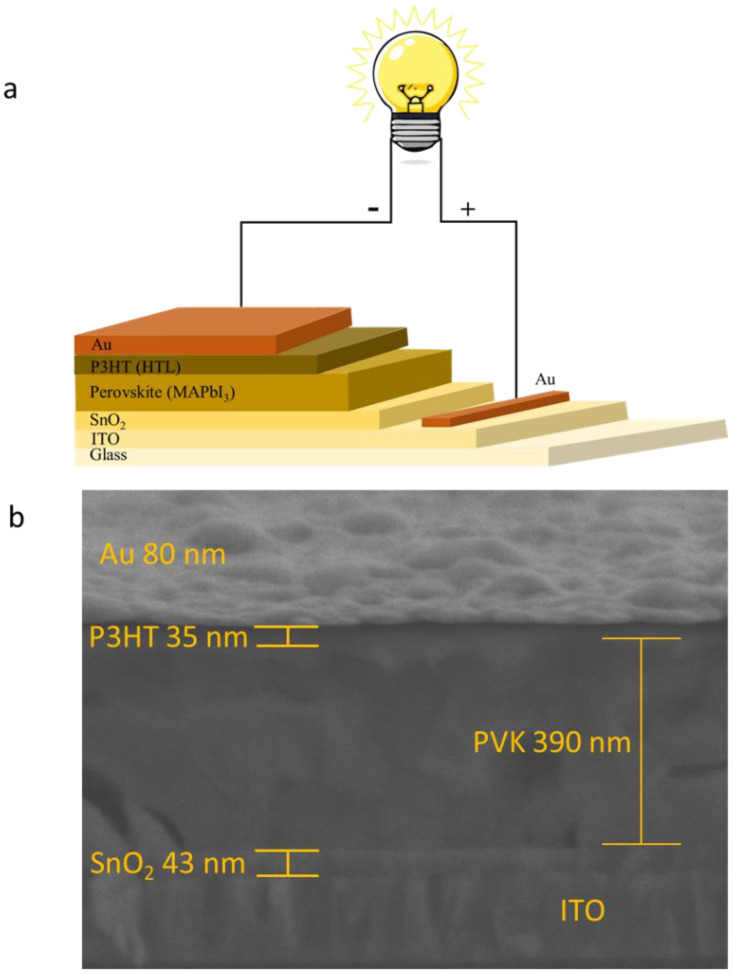

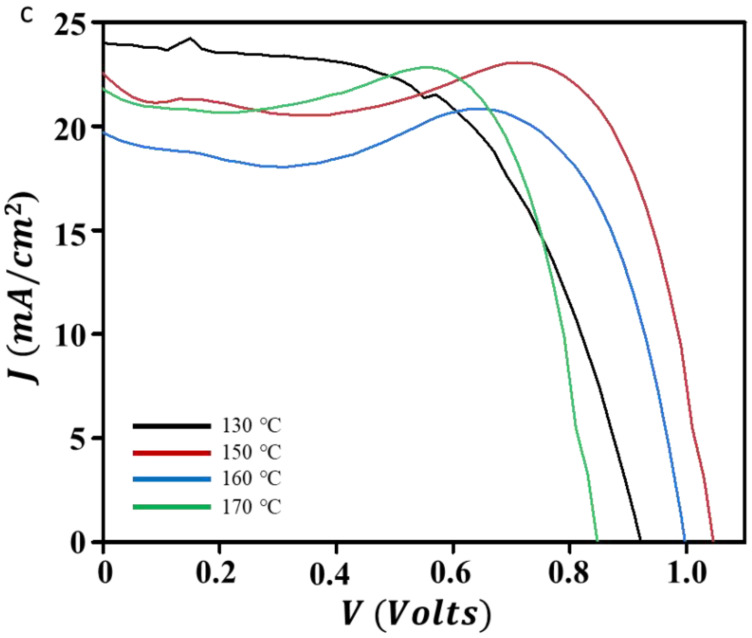
The perovskite solar cells configuration (**a**) and the cross-section image of the real PSC device (**b**). (**c**) J-V profile of the perovskite solar cells.

**Figure 2:**
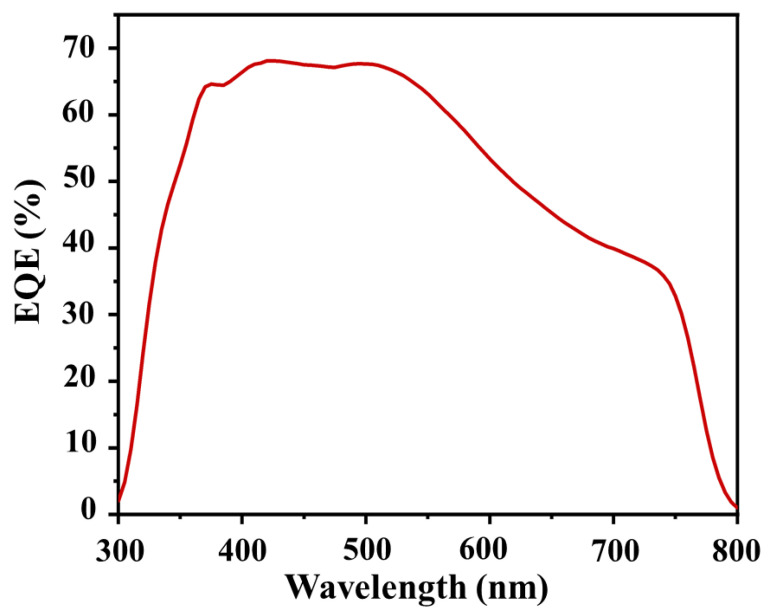
The typical external quantum efficiency spectrum of the champion device.

**Figure 3:**
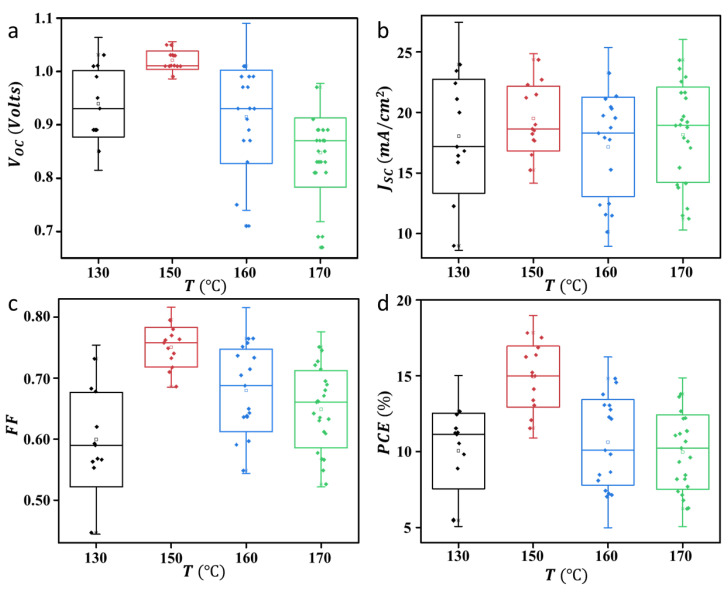
Statistical results of PSC photovoltaic parameters. (**a**) Voc, (**b**) Jsc, (**c**) FF and (**d**) PCE.

The perovskite solar cells with the ITO/SnO_2_/perovskite/P3HT/Au structure (see Figure 1a) have been fabricated to see how the perovskite MAPbI_3_ crystallinity properties, obtained via annealing treatment, can affect the performance of solar cells. The device cross-section image is shown in Figure 1b. Figure 1c is the typical J-V curve of the perovskite solar cells prepared using perovskite annealed at several different temperatures, namely 130, 150, 160, and 170 °C. It was found that the J-V response of the perovskite solar cells showed a strong dependence on the annealing treatment, where the values of *J*_sc_ and V_oc_ increase with increasing annealing temperature and are optimum for samples annealed at 150 °C. In this device, the values of *J*_sc_ and V_oc_ are 22.27 mA/cm^2^ and 1.05 V, respectively. With a fill factor (FF) value as high as 0.76, the power conversion efficiency of this device is 17.77%. This performance is amongst the highest for the PSC device prepared in the ambient condition. However, even though the *J*_sc_ value did not show a significant change with the perovskite annealing treatment, the device V_oc_ value decreased if the annealing temperature was increased to 160 and 170 °C. Perovskite’s lattice massive reconstruction might occur during this condition. Nevertheless, the J-V characteristic obtained in this study is fluctuating near the maximum power point toward the open circuit condition. This is due to the existence of high-trap density in the device that is induced by the humidity presence during the preparation of the device. This makes carrier extraction at the interface become unstable. We analyzed the external quantum efficiency (EQE) of the device by taking different devices with comparable power conversion efficiency. The result is shown in Figure 2. As the result reveals, the EQE spectrum indicates that there is a quite significant loss of the photocurrent at the region of 550 to 750 nm during the photovoltaic process. This is associated with a loss related to diffusion issues, such as low diffusion length that causes carrier transport instability, which can be attributed to the existence of the perovskite hydration at the grain boundary surface. Figure 3 shows the statistical analysis of perovskite solar cell performance obtained in this study, where the average value of *J*_sc_, V_oc_, FF, and PCE for champion devices are as high as 19.50 mA/cm^2^, 1.02 V, 0.75, and 14.92%, respectively. Table 1 summarizes the photovoltaic performance of the PSC under different perovskite crystallinity properties.

**Figure 4:**
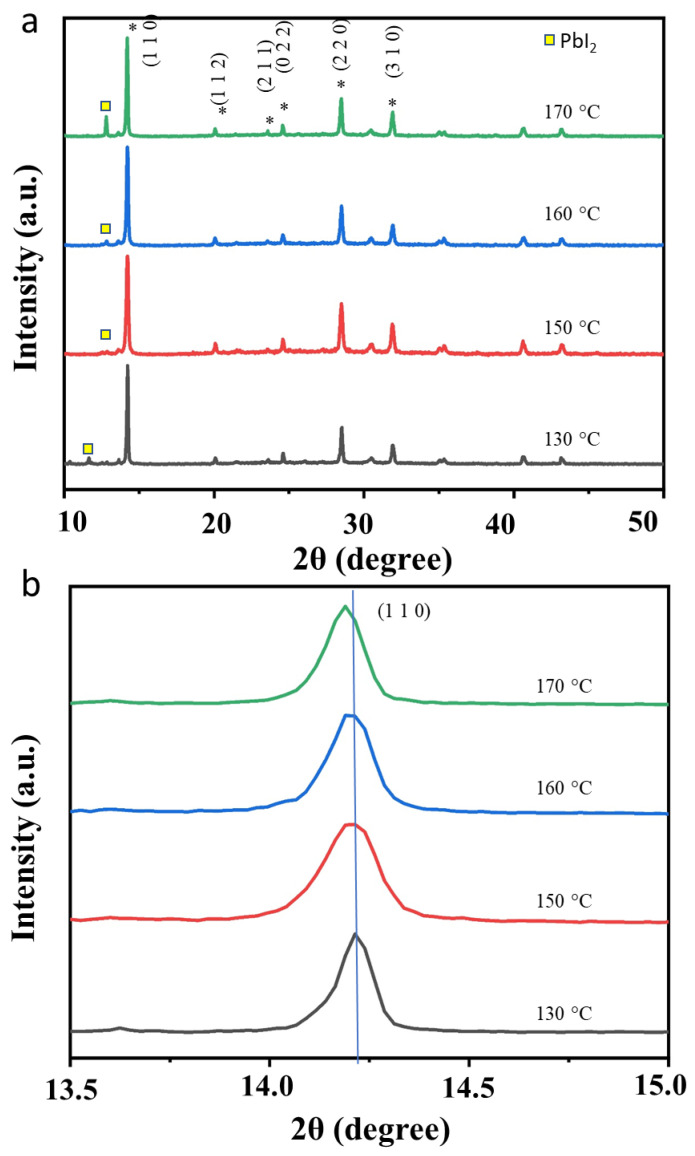
XRD pattern of the perovskite annealed at different temperatures, i.e., 130, 150, 160, and 170 °C. (**a**) wide scan. (**b**) zoom-in spectra at (110) plane.

**Figure 5:**
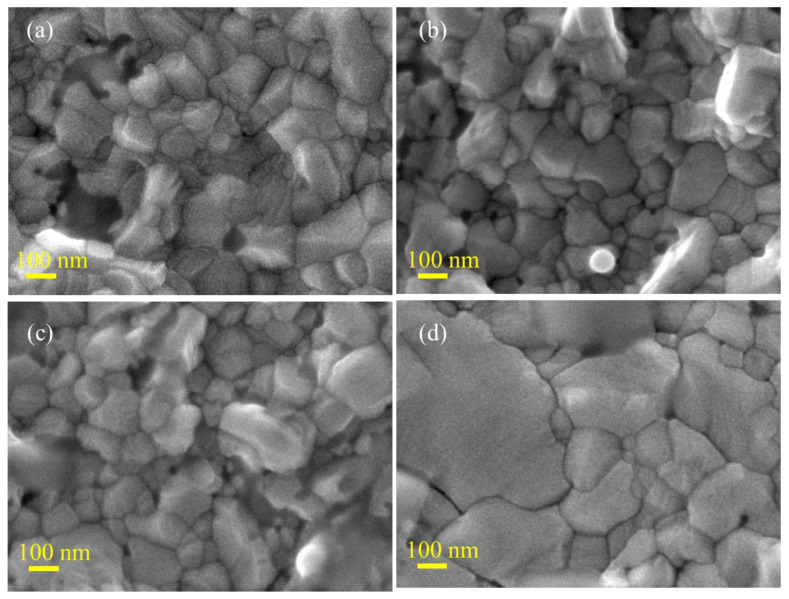
FESEM image of the perovskite samples annealed at 130 (**a**), 150 (**b**), 160 (**c**), and 170 °C (**d**).

We assume that the increase in the performance of the perovskite solar cells caused by variations in the annealing temperature is due to changes in the crystallinity properties of the perovskite, which involve refinement in the phase crystallinity or an increase in crystallite size [18,19,20,21]. To prove the existence of this process, we performed an X-ray diffraction spectroscopy analysis on the perovskite samples. The results are shown in Figure 4. In principle, the XRD pattern profile of each sample is the same and it corresponds to the standard diffraction pattern for the MAPbI_3_ perovskite (file no JCPDS 01-084-9474), i.e., tetragonal crystal system in *I4*/*mcm* space group with a crystal lattice *a* = 8.8548 and *c* = 12.6592. However, as the figure shows, it is observed that there is a slight difference in the intensity of the diffraction peak, especially peak (110), where the peak’s intensity was a little improved when the annealing temperature increased from 130 to 150 °C. It was then relatively unchanged when the annealing temperature was further increased above 150 °C. As the increasing peak intensity indicates an increase in the perovskite crystallite grain size, it can be remarked that the refinement in the phase crystallinity, when the sample annealing temperature increased from 130 to 150 °C, should exist. The Scherrer analysis further reveals that there is an increase in the crystallite size when the annealing temperature is increased (see Table 1). The FESEM analysis results further verify this process (Figure 5). We then analyze the position of the (110) peak to further understand the nature of the phase crystallinity refinement in this process. It is found that the peak’s position is gradually shifted to the lower angles with the increasing annealing temperature (Figure 4b). This infers that the refinement process occurs involving the expansion of the crystal lattice. Thus, enhanced optoelectrical properties are expected. 

**Figure 6:**
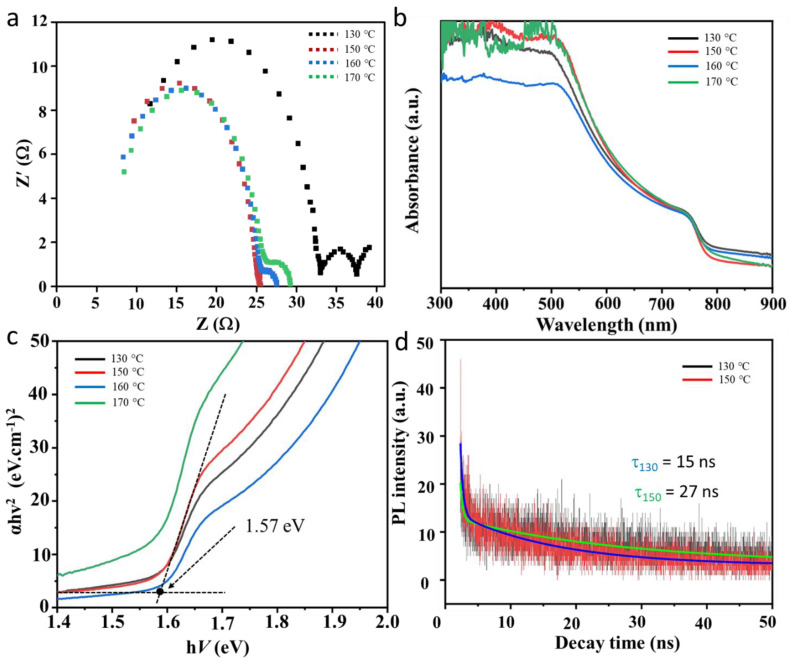
Optoelectrical properties of the perovskite solar cells under different crystallinity properties. (**a**) Electrochemical impedance spectra under 100 mWatts/cm^2^ light irradiation. (**b**) optical absorption spectra. (**c**) Tauc plot analysis of the sample’s band gap. Optical energy band gap calculation for the sample annealed at 150 °C is shown. (**d**) Transient photoluminescence spectrum of the champion sample compared with the sample annealed at 130 °C.

We then performed a FESEM analysis to perceive how the annealing temperature changes the crystallite’s morphology and size of the perovskite film. The results are shown in Figure 5. In good agreement with the XRD analysis result (Figure 4), the annealing treatment has significantly changed the perovskite grain size and also the grain morphology. For example, if a sample is annealed at a low temperature, namely 130 °C, the perovskite grains look quite compact without the presence of a clear grain boundary. The grain size is estimated at 200 nm. However, in this sample, the grain morphology is not very crystalline and shows an irregular morphology. While unnoticeable, the grain boundaries may enable efficient intragrain charge transfer, and non-crystalline shapes will affect interfacial charge transfer with different layered materials in the device. The grain morphology looks defined and has a higher shaped crystallinity when the annealing temperature is increased to 150 °C. In this condition, we observed that the grain boundary is invincible. The grain size increases slightly when compared to the sample annealed at 130 °C, which is around 250 nm. Furthermore, when the annealing temperature is further increased, such as to 160 and 170 °C, very significant changes are obtained. Although there is an increase in grain size, the shape morphology becomes more irregular with increasing annealing temperatures. In addition, the grain boundary increases with increasing annealing temperatures. It is expected that decreasing the crystallinity shape and increasing the grain boundaries will reduce the interfacial charge transfer and transportation dynamics in the device [10,22,23]. Therefore, as explained above in Figure 3, it can be concluded that the samples annealed at 150 °C will give the best performance due to the high crystallinity shape and invincible grain boundary, which results in highly energetic interfacial charge transfer and transportation in the device [24]. The electrochemical impedance spectroscopy (EIS) results seem to confirm this assumption (Figure 6a). Samples with a higher degreed crystallinity shape produce a greater interfacial charge transfer activity as indicated by the device’s low charge transfer resistance (*R*_ct_), namely 19.99 Ω in the champion device. Due to this highly active interfacial charge transfer, it has finally promoted a massive carrier extraction within the device, which is indicated by the high recombination resistance (R_rec_) of the champion device (see Table 1). According to the EIS analysis result, the recombination resistance of the champion device is lower than the sample annealed at 130 °C.

To prove the relationship between the crystallinity properties of the perovskite and the optoelectrical activity properties, we performed an optical absorption spectroscopy analysis. Figure 6b shows the optical absorption spectrum of the perovskite thin films annealed at different temperatures. As shown in the picture, at a glance the optical absorption profile of all the samples is the same with the absorption band edge at 780 nm. There is a slight difference in the optical density of the sample in the absorption region of the perovskite. This result indicates a difference in the optoelectrical properties of the perovskite when annealed at different temperatures. To acquire a clearer picture of the relationship between the optoelectrical properties and annealing temperatures, the optical energy band edge is evaluated by Tauc analysis. In addition to obtaining an estimate of the energy band edge of the perovskite material, a Tauc plot can also provide information on the optoelectrical dynamic of the perovskite [25]. The Tauc plot of the perovskite thin film sample is shown in Figure 6c. As shown in Figure 6c, the energy band edge of the perovskite film ranges from 1.55 to 1.58 eV. This value is indeed a normal value for the MAPbI_3_ perovskite. The difference in the band edge energy value is not only caused by the annealing temperature but also by other factors, such as film quality and stoichiometric condition of the perovskite. The optoelectrical dynamics are represented by the steepness of the curve near the energy band edge. A steep curve indicates that the carrier excitation density is high. Figure 6c shows the sample that was annealed at 150 °C is the sample that has the steepest curve. In other words, this sample has the best optoelectrical dynamic and will create the perovskite solar cells with the best performance. Normally, the device’s external quantum efficiency (EQE) analysis is also used to verify the effect of energy band gap modification on the device’s performance improvement. However, the optical absorption and photoluminescence analysis results are sufficient evidence that the change in the optical band gap due to annealing is the key origin of the device’s improvement. 

**Figure 7:**
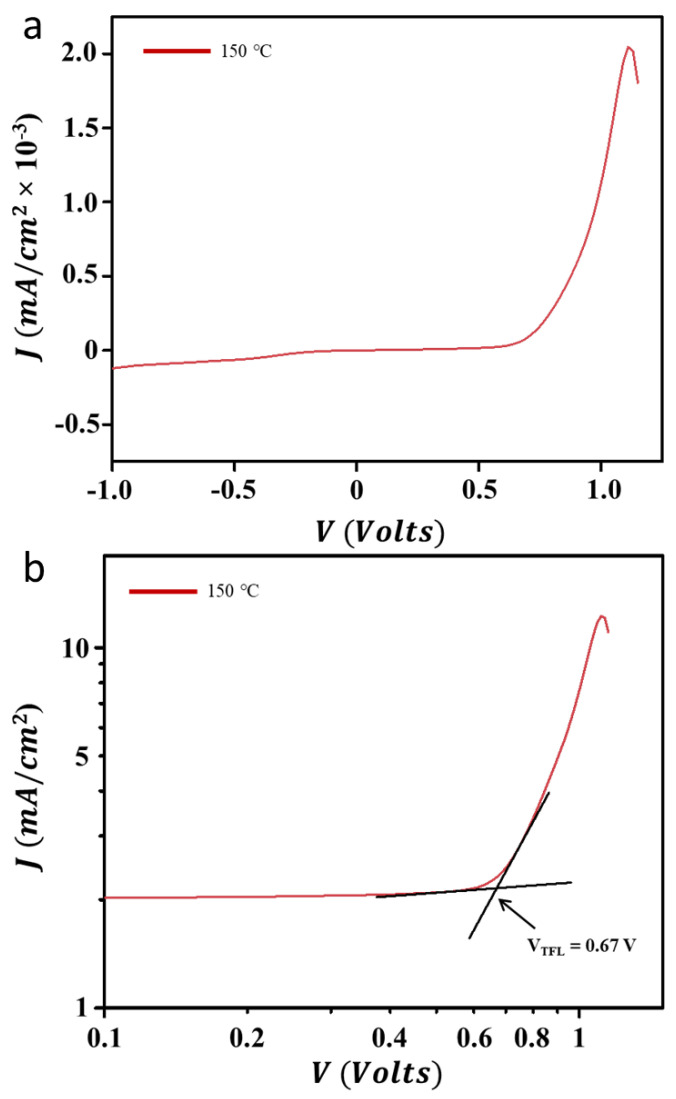
Carrier trap density analysis of the device. (**a**) Dark J-V curve and (**b**) double log J-V curve of the champion device.

We then performed a transient photoluminescence spectroscopy analysis on the champion sample and compared it with one of the lower-performance samples, namely a sample annealed at 130 °C, to show how the crystallinity properties of the perovskite film, which is related to the annealing temperature, affects its optoelectrical dynamics. The results are shown in Figure 6d. The transient photoluminescence curve is represented by the decay function curve which gives an overview of the lifetime of the electronic state in the excited state. In a perfect material system, the lifetime will be long and when used in perovskite solar cells, it will maintain a large photocurrent density [26]. The transient PL curve can be matched with the second-order decay function to achieve the lifetime of the electronically excited state. It was found that the champion sample had an excited state lifetime of almost two times longer (i.e., τ = 27 ns) when compared to the perovskite sample which was annealed at 130 °C (τ = 15 ns). Of course, it was to be expected that the champion sample’s carrier lifetime would be much larger when compared to other samples, such as samples that annealed at 160 and 170 °C. These results are certainly a solid reason as to why samples annealed at 150 °C produced the best power conversion efficiency. The transient PL curve is a little bit noisy, which could be due to the equipment’s resolution limit. However, curve fittings using a second-order decay function can finely show the decay trend and predict the carrier lifetime. We then carried out a dark J-V analysis (Figure 7a) to predict the carrier trap density of the device. By using a log-log dark J-V curve (Figure 7b), the carrier (electron and hole) trap density (N) can be estimated using the relation of N = 2 ε ε_0_ V_TFL_/e L^2^, where V_TFL_ is the trap filling limit voltage (see Figure 7b), ε and ε_0_ are the MAPbI_3_ perovskite permittivity (~65) and vacuum permittivity, respectively, e and L are electronic charges and the perovskite thickness (~390 nm), respectively. From these analyses (Table 1), it is found that the champion device exhibits the lowest carrier trap density, i.e., (3.18 × 10)^22^ cm^−3^. This verifies that the annealing treatment refines the crystallinity properties of the perovskite sample, modifying the trap density for enhanced carrier dynamics in the device. Thus, improving the power conversion efficiency of the perovskite solar cells. An electron-only or hole-only device should indeed be used to predict electron or hole-trap density. However, in most case, both the electron and hole density has a similar trap density value. Thus, the present calculation should be acceptable for the carrier trap density estimation.

Annealing treatment has been well-known to improve the crystallinity properties of perovskite materials. While ambient conditions during the annealing treatment influence the crystallinity refinement process, the effect of high humidity on the perovskite crystallinity properties is not well-understood at the moment. In recent literature, it is reported that annealing the perovskite humid condition, ca.~40% of relative humidity has successfully enlarged the perovskite grain size [27]. The presence of vapor molecules during thermal annealing treatment might control the liquidity of the perovskite, allowing a proper grain growth process. Annealing under higher RH, as in the present study might produce a better liquidity perovskite system for a better refinement process, a process that has been verified by XRD (Figure 4) and FESEM (Figure 5) analysis results. Nevertheless, while the high RH condition facilitates better liquidity properties for the refinement process, the perovskite hydration might also be dominant, generating surface roughening and trap. Further treatment to remove the excess hydrate on the perovskite surface, such as via vacuum annealing, is necessary to obtain high-performance, humid stable perovskite solar cells.

Indeed, the performance of perovskite solar cells in this study is still low compared to what has been reported previously and the effect of phase crystallinity refinement is also still inferior when compared to the use of other techniques to increase the performance of perovskite solar cells, such as composition engineering, etc. However, from this study, how the effect of annealing on the crystallinity properties and its effect on the optoelectrical dynamic properties of perovskite can be known. Continuous efforts to obtain more optimum conditions to adjust the crystallinity properties of perovskite under humid environments, make it possible to realize perovskite solar cells with high performance prepared in ambient conditions. As also presented in the result, the humidity conditions strongly affect the crystallinity properties of the hybrid perovskite. Annealing the perovskite under different humidity conditions may produce unique crystallinity properties of the perovskite, which in turn influence its performance in the application. We are pursuing this analysis to obtain a broad picture of what extent effect of humidity in the hybrid perovskite behavior. 

## 4. Conclusions

The relationship between the crystallinity properties of perovskite and its optoelectrical properties has been studied by changing the crystallinity condition of the perovskite thin film through annealing treatment. It has been found that the annealing temperature can determine the state of perovskite crystallinity, such as grain size, inter-grain interface, and grain morphology. In this study, the temperature of 150 °C was found to be the most suitable for obtaining perovskite films with a crystalline shape grain morphology as well as compact inter-grains with an invincible inter-grain interface. The grain size is about 250 nm. Although a higher annealing temperature can produce a large grain size, it has a non-crystalline morphology. In addition, the inter-grain interface is large enough that it will produce a high sheet resistance or will induce a phase change in the perovskite. The perovskite solar cells analysis showed that samples annealed at 150 °C produced the highest power conversion efficiency, namely 17.77%, much greater when compared to other samples. The photoluminescence spectroscopy studies show that samples annealed at 150 °C have a carrier lifetime two times longer than other samples. The electrochemical impedance spectroscopy studies also show that the champion device has highly energetic interfacial charge transfer caused by the crystalline shape grain morphology. These two facts are of course the main reason why samples annealed at 150 °C produce the highest power conversion efficiency. The effect of temperature on the material will indeed change the crystalline properties of the perovskite material. However, it is strongly influenced by the circumstances of the sample preparation. This study was carried out in an ambient environment with a relative humidity of 70%. The perovskite’s behavior under annealing treatment in high humidity conditions presented in this study can be a guide for obtaining perovskite solar cells with high performance in industrialization processes.

## Figures and Tables

**Table 1:** Photovoltaic parameters value of the PSC prepared from the perovskite with different crystallinity properties.

Sample	$V_{OC}$ (V) Max (avg ± sd)	$J_{SC}$ (mA/cm^2^) Max (avg ± sd)	FF Max (avg ± sd)	PCE (%) Max (avg ± sd)	$R_{S}$ (Ω)	$R_{ct}$ (Ω)	$R_{rec}$ (Ω)	N ($cm^{3}$)	Crystallite Size (nm)
130 °C	0.93 (0.94 ± 0.06)	23.96 (18.04 ± 4.70)	0.57 (0.60 ± 0.08)	12.70 (10.17 ± 2.49)	7.24	25.04	7.07	3.94 × 10^22^	537.7
150 °C	1.05 (1.02 ± 0.02)	22.27 (19.50 ± 2.67)	0.76 (0.75 ± 0.03)	17.77 (14.92 ± 2.02)	5.30	19.99	3.62	3.18 × 10^22^	561.6
160 °C	0.99 (0.92 ± 0.09)	19.55 (17.16 ± 4.10)	0.77 (0.68 ± 0.07)	14.90 (10.74 ± 2.82)	5.88	19.14	2.82	3.84 × 10^22^	578.9
170 °C	0.85 (0.85 ± 0.06)	21.62 (18.15 ± 3.93)	0.75 (0.65 ± 0.06)	13.78 (10.03 ± 2.45)	6.77	13.45	1.42	3.89 × 10^22^	781.7

## Data Availability

Data related to this research is available from the authors upon request.
